# The semantics of acute limb ischemia

**DOI:** 10.1590/1677-5449.202101451

**Published:** 2023-12-04

**Authors:** Guilherme de Castro-Santos

**Affiliations:** 1 Universidade Federal de Minas Gerais – UFMG, Faculdade de Medicina, Departamento de Cirurgia, Belo Horizonte, MG, Brasil.; 2 Hospital das Clínicas UFMG, Serviço de Cirurgia Vascular, Belo Horizonte, MG, Brasil.

Dear Editor,

It was very interesting and stimulating to read the article “Acute upper limb arterial ischemia in patients diagnosed with COVID-19: case series.”^1^ It is clear that this disease has a strong vascular component, which directly affects the areas of practice of angiologists and vascular surgeons. Presentation in upper limbs is less common, but of no less importance. However, the title of the article attracts attention in its use of a term that is probably redundant. In practice, we observe that all ischemia is arterial, unless there is *phlegmasia cerulea dolens*. Even in this case, if we analyze it in depth, we will also observe that ischemia is caused by the abrupt reduction of blood perfusion via the arterioles caused by severe venous stasis in the venules. In chapter 100 of the sixth edition of “Rutherford’s Vascular Surgery and Endovascular Therapy”, we find the term acute limb ischemia. ^2^ Similarly, in the books “Vascular Diseases for the Non-Specialist, ^3^ by professor Navarro, and “Doenças Vasculares Periféricas” [“Peripheral Vascular Diseases”], by professor Maffei, ^4^ we observe the terms “acute limb ischemia” and “oclusões arteriais agudas” [“acute arterial occlusions”]. None of them use the term “acute arterial ischemia” [“isquemia arterial aguda”]. It is necessary to differentiate between the terms “acute arterial occlusion” and “acute limb ischemia”. When we use the term “occlusion”, it could refer to the venous system or the arterial system, hence the need to make the site of occlusion explicit. In contrast, in the term “acute limb ischemia”, it is already implicit that the ischemia is arterial*.* This term is being increasingly used in the international literature rather than acute arterial occlusion. This is only a matter of semantics and in no way detracts from the work of our brilliant colleagues from São Paulo.
