# Supplementary material for: The semantics of acute limb ischemia
Source: J Vasc Bras. 2023 Dec 4;22:e20210145. doi: 10.1590/1677-5449.202101451 (PMC10878674; doi:10.1590/1677-5449.202101451)
Supplement: Translation

# Translation: A semântica da isquemia aguda de membros

**DOI:** 10.1590/1677-5449.202101452

**Authors:** Guilherme de Castro-Santos

**Affiliations:** 1 Universidade Federal de Minas Gerais – UFMG, Faculdade de Medicina, Departamento de Cirurgia, Belo Horizonte, MG, Brasil.; 2 Hospital das Clínicas UFMG, Serviço de Cirurgia Vascular, Belo Horizonte, MG, Brasil.

Caro Editor,

Muito interessante e instigante o artigo “Isquemia arterial aguda de membros superiores em pacientes diagnosticados com COVID-19: série de casos”^1^. Percebemos que essa doença tem um forte componente vascular, o qual afeta diretamente a área de atuação do angiologista e do cirurgião vascular. A apresentação em membros superiores é menos comum, mas não menos importante. Entretanto, o título chamou a atenção por usar um termo que seria provavelmente redundante. Na prática, observamos que toda isquemia é arterial, a menos que haja *flegmasia cerúlea dolens*. Nesse caso, se avaliarmos a fundo, também observaremos que a isquemia foi causada pela diminuição abrupta da perfusão sanguínea pelas arteríolas em decorrência de uma estase venosa grave nas vênulas. Na sexta edição do Rutherford’s Vascular Surgery and Endovascular Therapy^2^, no capítulo 100, observamos o termo *acute limb ischemia*. Da mesma forma, nos livros Vascular Diseases for the Non-Specialyst^3^, do professor Navarro, e Doenças Vasculares Periféricas, do professor Maffei^4^, observamos os termos “isquemia aguda de membros” e “oclusões arteriais agudas”. Em nenhum deles há o termo “isquemia arterial aguda”. É necessário diferenciar o termo “oclusão arterial aguda” de “isquemia aguda de membros”. Quando usamos o termo oclusão, este pode ser usado tanto para o sistema venoso como o arterial, daí a necessidade de explicitar o local da oclusão. O termo “isquemia aguda de membros” já carrega em si, implicitamente, que a isquemia é arterial*.* Esse termo tem sido cada vez mais usado na literatura internacional quando comparado ao termo oclusão arterial aguda. Trata-se somente de uma questão de semântica, um detalhe que de forma alguma desmerece o trabalho dos brilhantes colegas de São Paulo.
