# Supplementary material for: The semantics of acute limb ischemia
Source: J Vasc Bras. 2023 Dec 4;22:e20210145. doi: 10.1590/1677-5449.202101451 (PMC10878674; doi:10.1590/1677-5449.202101451)
Supplement: Related texts

# Reply: RESPONSE LETTER

**Authors:** Felipe Damascena Rosa, Marcelo Calil Burihan, Elexandra Aparecida Simões, João Paulo de Souza Abdala, Orlando da Costa Barros, Felipe Nasser

**Affiliations:** 1 Hospital Santa Marcelina, São Paulo, SP, Brasil.; 2 Hospital Israelita Albert Einstein, São Paulo, SP, Brasil.

Dear Editor,

After reviewing the consolidated literature, especially the most recent editions of the books mentioned in the letter, we conclude that the term “arterial ischemia” is indeed redundant. Perhaps because of its routine use in clinical practice, and intending to stress that the ischemia was of arterial origin, we (both the authors and the reviewers) failed to notice this error of language use that was well spotted by our colleague.

# Reply: RESPOSTA

**Authors:** Felipe Damascena Rosa, Marcelo Calil Burihan, Elexandra Aparecida Simões, João Paulo de Souza Abdala, Orlando da Costa Barros, Felipe Nasser

**Affiliations:** 1 Hospital Santa Marcelina, São Paulo, SP, Brasil.; 2 Hospital Israelita Albert Einstein, São Paulo, SP, Brasil.

Prezado Editor,

Após revisão da literatura consolidada, especialmente das edições mais recentes dos livros citados, concluímos que o termo “isquemia arterial” é, de fato, uma redundância. Talvez pelo uso corriqueiro na prática clínica, e no ímpeto de enfatizar que a isquemia era de origem arterial, tenha nos passado despercebido (tanto aos autores quanto aos revisores) o vício de linguagem muito bem apontado pelo colega.
